# Cocktail Sausage Supplemented With Whole Tomato Powder Encapsulated in Chia Seed Mucilage ( *Salvia hispanica* L.) by Lypholization: The Color, Sensory, Textural Properties, and Oxidation Stability of Sausage

**DOI:** 10.1002/fsn3.4594

**Published:** 2024-11-25

**Authors:** Seyed Saeed Sekhavatizadeh, Fatemeh Faryabi, Mohammad Ganje

**Affiliations:** ^1^ Fars Agricultural and Natural Resources Research and Education Center, AREEO Shiraz Fars Iran; ^2^ Department of Food Science and Technology Bushehr Institute of Kherad Higher Education Bushehr Iran; ^3^ Department of Agriculture, Minab Higher Education Center University of Hormozgan Bandar Abbas Iran

**Keywords:** chia seed mucilage, cocktail sausage, encapsulation, lyophilization, tomato powder

## Abstract

The deterioration of meat products is significantly influenced by the oxidation of lipids. The addition of antioxidants is one of the accepted methods to retard lipid oxidation. The goal of this research was to encapsulate tomato powder with chia seed mucilage by lyophilization. Tomato powder and chia seed mucilage were used as the wall material at three ratios 1:1 (T1:C1), 2:1 (T2:C1), and 1:2 (T1:C2). The particle size, encapsulation efficiency, rheology, and solubility index of the beads were assessed. Three sausage samples, including the control, 3% w/w tomato powder (TP_sample_), and 6% w/w bead (EnTP_sample_), were produced. The color, texture, peroxide, thiobarbituric acid, and sensory parameters of the sausage were analyzed during storage. The results showed that T2:C1 had a maximum encapsulation efficiency (44.71%) with particle size (172.31 nm). T1:C1 had a highly significant value in the solubility index (90.09% w/w), but for the viscosity parameter, T1:C2 had a maximum value among the samples. FTIR and X‐ray diffraction analyses demonstrated successful encapsulation in all samples. The water holding capacity (6.92% w/w), hardness (2992.5 g), and gumminess (2772.3 g) were the highest, but cooking loose (10.98% w/w) the lowest in the EnTP_samples_. Higher color and odor scores were recorded for the EnTPs. In addition, the encapsulated tomato powder had a significantly (*p* < 0.05) lower peroxide value (7.34 mEq/kg) and thiobarbituric acid (2.09 mg MDA/kg) in the sausages than in the other samples. In conclusion, the incorporation of EnTP_sample_ as a polyphenol component in cocktail sausages is more advantageous than TP alone.

## Graphical abstract

The tomato bead (T2:C1) had maximum encapsulation efficiency with low particle size. The water holding capacity, hardness, and gumminess were the highest, but cooking loose lowest in the EnTP_samples_. The incorporation of EnTP_sample_ as a polyphenol compound in cocktail sausages is more advantageous than TP alone.

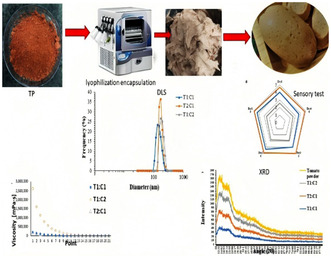

## 1 Introduction

During the production of processed meat products, exposure to a variety of contaminants is unavoidable. As a result, the presence of these contaminants often leads to both economic challenges and hygiene problems (Ataş et al. 2021). The conservation of processed meat products involves addressing the significant challenge of minimizing lipid oxidation. It can cause color and texture changes, decrease the shelf life, and produce a rancid flavor that leads to reduced acceptability of these food products by consumers. Meat‐based products that are prepared as ready‐to‐eat food, such as salami and sausages, not only serve as healthy and nutritious foods but also boast a fat content of up to 3% w/w based on the specific formulation (Pamuk, Gedikoğlu, and Sökmen 2022).

For many years, synthetic antioxidants have served as effective agents for preventing oxidation in various foods, including meat and meat products. The compounds that are predominantly applied in food as antioxidants include butylated hydroxytoluene (BHT), tert‐butylhydroquinone (TBHQ), octyl gallate (OG), propyl gallate (PG), and butylated hydroxyanisole (Bashir et al. 2024). Synthetic antioxidants are increasingly used as effective agents. They are replaced with natural antioxidants due to their accumulation in the human body, which can potentially cause disease or allergies (Hadidi et al. 2022). The potential for using plant extracts as natural antioxidants with various capacities in meat products should be considered (Pamuk, Gedikoğlu, and Sökmen 2022). One of these plants is tomato. Tomatoes are extensively consumed worldwide, both in fresh or processed form (canned tomatoes, sauces, juices, ketchup, soups, etc.). The bioactive substances and nutrients in this fruit are significant. In humans, it can prevent or reduce various chronic degenerative diseases and is beneficial for weight loss, healthy skin, and a healthy body (Ganje et al. 2024).

Tomato by‐products have been integrated into diverse food formulations, such as plant‐based meat products (i.e., patties and meat‐free sausage), extruded puffed snacks, and meat products, as well as bread. The presence of dietary fibers in food products often leads to positive effects on their nutritional quality and color, as well as causes changes in their textural quality (Lyu et al. 2024).

Changes in natural antioxidant structure and composition caused by various factors, including temperature, oxygen, light, and pH can limit the use of these substances in food. As a result, the addition of natural antioxidants to food results in a reduction in antioxidant activity when the food is subjected to high temperatures (Sekhavatizadeh et al. 2023).

Microencapsulation can be used to maintain the antioxidant activity of plant extracts. Microencapsulation is particularly well suited for delivering high‐value compounds, as it can stabilize and regulate the release of effective ingredients extracted from fruits and vegetables or their derivatives. Additionally, this technique enhances the shelf life, leading to improved final quality of product characteristics and the potential to enhance the bioavailability of each active compound through release mechanisms in the gastrointestinal tract. Moreover, microencapsulation reduces the negative effects of incompatible compounds, moisture, and oxygen in food (Borhanpour et al. 2022). Several microencapsulation techniques, such as extrusion, spray drying, and emulsion, exist (Sekhavatizadeh et al. 2024). Another encapsulation technique that can be employed is lyophilization, which has a protective effect on the phenolic compounds present in food and allows it to be maintained for increasing storage time (Lopez‐Polo et al. 2020).

The presence of various wall materials, including lipids, proteins, and polysaccharides, in the encapsulation of polyphenols induces significant characteristics, such as resistance to thermal degradation, stability to oxidation, and control release. These features have been consistently reported in numerous studies (Sekhavatizadeh et al. 2023). The process of food encapsulation should be conducted using food‐grade materials that are suitable for human consumption.

Additionally, it is essential that all wall substances do not react with the core material, enable quick drying, and are cost‐effective. The preservation of volatile compounds, low solubility, and viscosity of polysaccharides make them desirable choices for this purpose (Zeidvand et al. 2024).

For this reason, in the present study, chia seed mucilage (CSM) was used for encapsulation as a wall material. The CSM is a heteropolysaccharide with a high molecular weight and complex structure. It is extracted when the seed absorbs water and leads to increasing solution viscosity. The produced mucilage gel containing 6% w/w soluble fiber formed a transparent capsule around CSM (Yüncü, Kavuşan, and Serdaroğlu 2022). The chia plant is classified as an herbaceous plant belonging to the *Lamiaceae* family (Pires et al. 2020). *Salvia hispanica* L. is an annual herbaceous and macrothermal short‐day flowering plant, native of southern Mexico and northern Guatemala. The species *S. hispanica* produces numerous dry indehiscent fruits, which are commonly called seeds. These small white and dark seeds in pre‐Columbian times, along with corn, beans, and amaranth, were one of the basic foods in the diet of several people. Chia is a macrothermal short‐day flowering plant (De Falco, Amato, and Lanzotti 2017).

The addition of chia to formulations has been found by researchers to increase nutritional value due to its high dietary fiber content, enhanced technological properties, and protection against lipid oxidation through the presence of phenolic compounds (Antonini et al. 2020). Furthermore, the structure of polysaccharides consists of dietary fibers with xylans (Timilsena et al. 2016). The ability of mucilage to crosslink and absorb 10–100 times its weight in water may be related to the unique structure of polysaccharides and their constituents. Additionally, mucilage contains hemicellulose, which contributes to oxygen and oil barrier properties (Yüncü, Kavuşan, and Serdaroğlu 2022).

The main goal of this research was to encapsulate tomato powder (TP) with CSM. For this purpose, (1) the phenolic compositions of TP and CSM were determined; (2) the physical properties of the beads consist of EE%, bulk density (BD), SI, X‐ray diffraction analyses (XRD), viscosity, and Fourier Transform Infrared Spectroscopy (FTIR), were assessed; and (3) the effect of the encapsulated TP on the quality parameters of the cocktail sausage (i.e., texture, hunter color values, proximate composition, pH, water‐holding capacity (WHC), peroxide value (PV), and thiobarbituric acid reactive substances (TBARS) was investigated during cold storage).

## 2 Materials and Methods

### 2.1 Chemicals

The chemicals, solvents, and standards included hydrochloric acid, sodium hexane‐sulfonate, methanol (HPLC grade), thymol, rosmarinic acid, eugenol, glacial acetic acid, hesperetin, ellagic acid, gallic acid, catechin, sinapic acid, trans‐ferulic acid, vanilin, carvacrol, p‐coumaric acid, coumarin, hesperidin, quercetin, caffeic acid, chlorogenic acid, hexan, formic acid, chloroform, potassium iodide, sodium thiosulfate, trichloroacetic acid, thiobarbituric acid, tri‐sodium phosphate, sodium nitrite, and other standard reagents were obtained from Merck (Darmstadt, Germany). Gallic acid was obtained from Acros Organics (New Jersey, USA). Corn oil was obtained from the Narges Oil Company, Shiraz, Fars, Iran. Garlic, soy protein isolate, gluten, starch, spice, and NaCl were obtained from Behinazma Co, Shiraz, Fars, Iran.

### 2.2 Raw Materials

Fresh‐type tomatoes (*Solanum lycopersicum* L., Var. *Brivio*) were selected on the basis of the following criteria: uniform size, fresh appearance, firmness, and insect‐free nature. Fresh meat was obtained from Behin Co, Marvdasht, Fars, Iran.

### 2.3 Mucilage Extraction

Chia mucilage was extracted from seeds using distilled water at a ratio of 1:30 (seeds to water) in a water bath at 50°C with stirring (1500 rpm) for 2 h. The collected mucus was then filtered, dried in an oven (overnight at 45°C), ground, and sieved through an 18 mesh sieve. The final dry powder was packaged and stored in a cool and dry place.

### 2.4 TP Preparation

To prepare TPs, fresh and ripe red tomatoes without any spots or damage were used. The tomatoes were adequately washed with clean drinking water and 1% w/v sodium chloride solution. Then, the tomato juice was extracted using an extractor (Moulinex XXL JU655, France). Next, the samples were frozen at a temperature of −35°C for 24 h and dried in a vacuum freeze dryer (Alpha 2‐4 LD plus, Christ, Germany) at −20°C for 24 h. The dried samples were packed in polyethylene bags and kept under normal conditions (Chauhan and Antarkar 2023).

### 2.5 Determination of Polyphenolic Compounds in CSM and TP

High‐performance liquid chromatography (HPLC) was used to determine polyphenolic compounds in CSM and TP. The system was equipped with a Zarbax eclipse (XDB) C18 column with a length of 150 m and an internal diameter of 4.6 mm, which was filled with 5‐μm particles, and the temperature of the column was 30°C with a UV detector at 280 nm. The flow rate was equal to 1 mL/min, and the volume of each sample injection was 20 μL. The mobile phase consisted of two parts, A (methanol) and B (1% v/v formic acid), according to Table 1 (Bahmanzadegan et al. 2019). The data were summarized using ChemStation software (Sekhavatizadeh, Hosseinzadeh, and Mohebbi 2021).

**TABLE 1:** HPLC gradient program for assessment of polyphenolic compound (Sekhavatizadeh, Hosseinzadeh, and Mohebbi 2021).

Time (min)	Flow rate (mL/min)	Composition	
		Mobile phase A (methanol)	Mobile phase B (Formic acid)
0	1	10	90
10	1	25	75
20	1	60	40
30	1	70	30
40	1	70	30

### 2.6 Microencapsulation Procedure

TP was used as the core material, and chia mucilage was used as the wall material in the three formulations according to Table 2. All materials were homogenized by a homogenizer at 12000 rpm for 2 min to form an aqueous solution that reached 12% w/v total solid soluble materials (Montiel‐Ventura et al. 2018). The prepared aqueous solutions were dried using a freeze dryer at −42°C and 10 Pa for 48 h, after which the beads were collected, sealed, and stored in a refrigerator at 4°C until further analysis (Gheonea et al. 2021).

**TABLE 2:** Formulation, BD, and SI of the beads contain TP and CSM.

Treatment	Formulation			BD (g/mL)	SI (%)
	Water	CSM (g)	TP (g)		
T1:C1	73	5	5	0.54 ± 0.04 a	90.09 ± 0.23 a
T2:C1	110	5	10	0.50 ± 0.02 a	80.07 ± 0.25 b
T1:C2	110	10	5	0.50 ± 0.04 a	70.08 ± 0.29 c

*Note:* Data (mean ± standard deviation) are from three replications. Means followed by different lowercase letters in row differ (*p* < 0.05) by Duncan test.

Abbreviations: BD, bulk density; CSM, chia seed mucilage; SI, solubility index; TP, tomato powder; T1:C1, TP:CSM ratio in bead wall formulation were 1:1; T1:C2, TP:CSM ratio in bead wall formulation were 1:2; T2:C1, TP:CSM ratio in bead wall formulation were 2:1.

### 2.7 Particle Size (PS) Distribution

To determine the average droplet size, a dynamic light scattering (DLS) instrument (SZ‐100, Horiba Ltd. Kyoto, Japan) was used. The target temperature was 25°C, and the scattering angle was 90°. One gram of beads was suspended in 100 mL of deionized water. Then, 1 mL of the suspension was inserted into a polycarbonate cuvette (with the opening of quartz glass cuvette 2). After bead dispersion, polystyrene latex with a refractive index of 1.3326 was chosen as the standard material. The SZ‐100 software for Windows was used for data analysis. For calculation, the standard mode was selected. The ND filter was adjusted automatically. The focus position in the center and the detector position at a 173° angle were adjusted.

### 2.8 Encapsulation Efficiency %

The EE% was measured based on the ratio of bioactive compound (gallic acid) in the beads before applying the encapsulation technique and in encapsulated sample according to Equation (1).

(1) $$\begin{array}{c} \text{Encapsulation efficiency}\;\left(\%\right)\\ \;=\frac{\text{Gallic acid}\;\left(\text{Encapsulated sample}\right)}{\text{Gallic acid}\;\left(\mathrm{TP}+\mathrm{CSM}\;\mathrm{as}\;\text{primary materials}\right)}\times 100 \end{array}$$

### 2.9 Bulk Density

BD was assessed by gradually adding 2 g of bead powder to a graduated cylinder with a capacity of 10 mL. Then, the volume was measured, and the BD was calculated by dividing the mass (g) by the volume (Montiel‐Ventura et al. 2018).

### 2.10 Solubility Index (SI)

To measure the SI of the finely coated TP (bead), an aqueous solution of bead sample (1% w/v) was incubated for 30 min in a shaker water bath at a constant temperature (25°C). Then, the solution was centrifuged at 2100 × *g* for 15 min. Ten milliliters of the supernatant were poured into a petri dish and dried in an oven with air circulation at 120°C for 4 h. The solubility of the sample was calculated based on Equation (2).

(2) $$\mathrm{WHC}\;\left(\%\right)=\frac{m2-m1}{m1}\times 100,$$

where m1 is the sample weight before centrifugation (*g*), and m2 is the weight of the sample after centrifugation (*g*).

### 2.11 Viscosity

For the assessment of viscosity and other rheological parameters, a rheometer (MCR 302, Anton Paar, Austria) was used. The prepared samples (10%, w/v _DW_) were left for 12 h before viscosity measurement. The sample (5 mL) was placed in concentric cylinder geometry at 23°C with a shear rate ranging from 10 to 100/s (Ganje et al. 2024).

### 2.12 X‐Ray Diffraction (XRD) Analysis

Crystal structure analysis was performed by X‐ray diffraction (XRD). The conditions for conducting this test were a voltage of 40 kV and a current of 30 mA. Bead powder samples were loaded on an aluminum plate, and X‐ray diffraction profiles were obtained in the range of diffraction angles (θ2) 10–80 with a scanning speed of 0.02/min.

### 2.13 Fourier Transform Infrared Spectroscopy

The infrared spectra of the TP, CSM, T1:C1, T2:C1, and T1:C2 samples were obtained to analyze the functional groups and to provide insight into the structural characteristics of the samples. The FTIR spectra were recorded with a Tensor II FTIR spectrometer (Bruker). All spectra were recorded at a wavelength of 400–4000 cm^−1^ (Fatemeh and Saeed 2019).

### 2.14 Preparation of Cocktail Sausages

To produce cocktail sausage (60% w/w meat), 3‐year‐old Holstein veal meat was used. The ingredients used to prepare cocktail sausage included meat, ice, wheat starch, gluten, soy protein isolate, spices, salt, and frozen garlic. TP in the free form and encapsulated form (EnTP) at concentrations of 3% w/w and 6% w/w, respectively were added to the sausage formulation (Table 3). In this study, cocktail sausage without TP or EnTP was produced as a control sample. To produce the samples, first, the red meat (veal) was ground by an electric grinder (Moulinex, Ecully, France) with a grid diameter of 4 mm. The quantities of the raw materials were weighed according to the formulation, and a mini‐cutter (Nova, Iran) was used to prepare the sausage emulsion (butter). Then it was transferred to the filling machine and filled in polyamide covers. Then, the packaged product was transferred to the cooking room using steam (78°C) for 1.5–2 h, and cooking was performed until the internal temperature of the product reached 70°C–72°C. Subsequently, the product was cooled with cold water to 20°C and stored at 0°C–4°C. The physicochemical and sensory properties were assessed during 40 days of storage at intervals of 10 days.

**TABLE 3:** Formulation of cocktail sausages prepared with different concentrations of TP and encapsulated form.

Ingredients (w/w %)	Treatments		
	EnTPsample	TPsample	Contsample
Beef	56.4	58.2	60
Corn oil	15.98	16.49	10
Tri sodium phosphate	0.47	0.485	0.5
Sodium nitrite	0.235	0.2425	0.25
Garlic	1.504	1.552	1.6
Soy protein isolate	1.88	1.94	2
Gluten	1.88	1.94	2
Starch	2.82	2.91	3
Spice	1.222	1.261	1.3
NaCl	0.94	0.97	1
Ascorbic acid	0.047	0.0485	0.05
Sodium caseinate	1.222	1.261	1.3
Ice powder	15.98	16.49	17
Beads	6.0	0	0
TP	0	3.0	0
Total	100	100	100

Abbreviations: CSM, chia seed mucilage; TP, tomato powder.

### 2.15 Proximate Value

The samples were analyzed for moisture and macronutrient contents (fat, ash, and protein) according to the methods described by Sekhavatizadeh, Hosseinzadeh, and Mohebbi (2021). The Macro‐Kjeldahl method (6.25 for sausage) (KjelFlex K‐360, Buchi, Flawil, Switzerland) was employed to measure the protein content. A Soxhlet apparatus was used to measure crude fat, and for the determination of ash content, samples were incinerated at 550°C ± 15°C (Elnaz and Saeed 2020; Sekhavatizadeh, Hosseinzadeh, and Mohebbi 2021).

### 2.16 Cooking Losses and Water Holding Capacity (WHC)

Cooking losses were determined by weighing individual samples before and after cooking and expressing weight loss as a percentage of the original weight.

To determine the water holding capacity, 10 g of sample was mixed with 15 mL of 0.6 M sodium chloride solution in a tube and then centrifuged at 4°C at 3000 × *g* for 15 min. The water holding capacity (WHC) was determined according to Equation (3):

(3) $$\mathrm{WHC}\;\left(\%w/w\right)=\frac{m2-m1}{m1}\times 100$$

where m1 is the sample weight before centrifugation (*g*), and m2 is the weight of the sample after centrifugation (*g*).

### 2.17 Texture and Color

The texture profile analysis (TPA) of the sausages was measured by Brookfield texture analysis (Model: CT3‐4500, Brookfield Engineering, USA) on the first and 40th day of the storage time. A TA7 cylindrical aluminum probe was used at a speed of 0.5 mm s^−1^ in compression mode, and a rupture distance of 1.0 mm was used. The peak force was measured in grams (Jouki, Shakouri, and Khazaei 2021).

To evaluate the sausage color parameters, a chroma meter (CR‐400, Konica Minolta, INC, Osaka, Japan) was used. A white tile was used for the calibration of the chromameter. The standard color parameters were L* = 23.92, a* = −1.29, and b* = 1.19. For each measurement, 5 g of sample was placed in a special chamber. The brightness component L*, redness tendency a*, and yellowness b* for each microsphere sample were measured. Color parameters were measured for each sample at different sites (Karimi, Sekhavatizadeh, and Hosseinzadeh 2021).

### 2.18 Peroxide Value

The peroxide value was assessed on the first and 40th day of the storage time. For fat extraction from sausage samples, a mixture of water, methanol, and chloroform at a ratio of 30:50:100 was used. Then, 1 g of extracted fat was mixed with 1 mL of saturated potassium iodide solution and placed in the dark for 10 min. Distilled water (30 mL) was added, and the mixture was mixed. Next, it was titrated with 0.01 M sodium thiosulfate‐containing starch solution. Finally, the amount of peroxide was measured according to Equation (4):

(4) $$\mathrm{PV}\;\left(\frac{\mathrm{meq}}{\mathrm{kg}}\right)=\frac{S\times N\times 100}{M\;},$$

where S is the titration volume, N is the normality of sodium thiosulfate (0.01 N), and M is the amount of fat extracted that is used (g).

### 2.19 Thiobarbituric Acid‐Reactive Substances

The TBARS was assessed on the first and 40th day of the storage time. For this purpose, 5 g of each sausage sample was mixed with 15 mL of trichloroacetic acid solution (20% v/v), homogenized for 1 min, and filtered through a Whatman filter paper (No. 1). Then, 2 mL of the solution was transferred to a test tube and mixed with 2 mL of 0.02 M thiobarbituric acid. Then, the mixture was incubated at 90°C for 30 min for the reaction. After cooling, the absorbance of the sample was recorded at a wavelength of 532 nm. Finally, the TBARS was measured in milligrams of malondialdehyde (MDA)/kg of sample.

### 2.20 Sensory Evaluation of Sausage

A panelist group consisting of 45 trained people performed the sensory evaluation through a 5‐point hedonic scale on the first and 40th day of the storage time. On the scale, five indicated “like extremely” and 1 “dislike extremely” in comparison with a control sausage. The participants were divided into two age groups: 19–25 years and 26–55 years (43% male and 57% female). Sausage samples were cut into thin slices 2–3 cm in length and heated in a microwave (MH8265CIS, LG, South Korea) for 15 s. Crackers without salt and water were given to the panelists in the interval between testing each sample. The characteristics of color, taste, smell, texture, and total acceptance were evaluated. Sensory evaluations were carried out from the first day of production at intervals of 10 days until the end of the storage period.

### 2.21 Statistical Analysis

The data were statistically analyzed using SPSS software (SPSS ver. 21.0). All the statistical analyses were performed using one‐way ANOVA and Duncan's multiple range test for the detection of mean differences among the samples (*p* < 0.05).

## 3 Results and Discussion

### 3.1 Determination of Polyphenolic Compounds in CSM, TP, and Beads

The polyphenolic compounds identified in TP and CSM by HPLC (are shown in Table 4). The results showed that rosmarinic acid (61.11 mg/L) was dominant in CSM, and gallic acid (7535.778 mg/L), was detected in TP. The gallic acid was not detected in CSM, but it was detected in TP. The percentages of gallic acid in T1:C1, T2:C1, and T1:C2 were 2018.57, 3369.0, and 1936.42 mg/L, respectively.

**TABLE 4:** Polyphenol content of TP, CSM.

Polyphenol content (mg/L)	CSM	TP	Retention time (min)
Gallic acid	ND	7535.778	3.3
Catechin	ND	ND	8.3
Chlorogenic acid	ND	42.6	10.5
Caffeic acid	ND	105	11.6
Vanilin	3.11	ND	13.5
p‐Coumaric acid	ND	9.79	15.6
Trans‐ferulic acid	ND	ND	16.3
Rosmarinic acid	61.11	ND	19.2
Quercetin	16.51	28.68	21.6

Abbreviations: CSM, chia seed mucilage; ND, not detected; TP, tomato powder.

### 3.2 PS Distribution

The size of the bead is important due to its influence on its physicochemical and functional properties, such as the appearance, texture, and survival ability of bioactive compounds (Choi and McClements, 2020). Moreover, it directly affects the release and absorption of bioactive compounds (Mahalakshmi et al., 2020).

**FIGURE 1:**
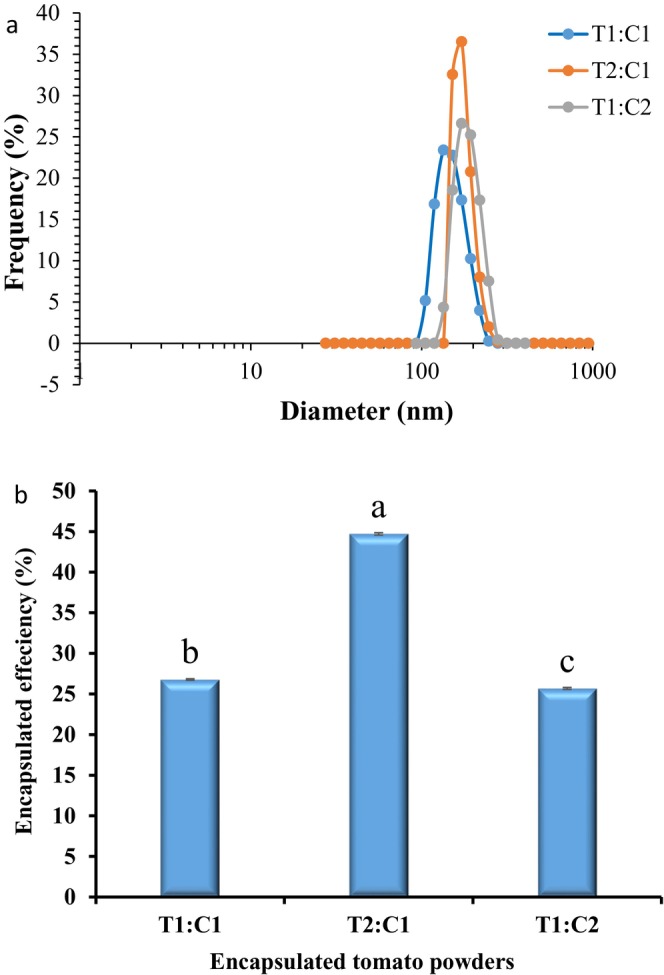
Size distribution (a) and encapsulation efficiency (b) of beads contain TP and CSM in different ratio as a wall material. T1:C1, TP:CSM ratio in bead wall formulation were 1:1; T2:C1, TP:CSM ratio in bead wall formulation were 2:1; T1:C2, TP:CSM ratio in bead wall formulation were 1:2. CSM, chia seed mucilage; TP, tomato powder.

The PS of the beads is shown in Figure 1a. An increase in the TP‐to‐CSM ratio led to PS enhancement. Therefore, the lowest PS in the T1:C1 sample was 134.16 nm. The creation of smaller particles increases the surface area per unit volume, leading to enhancements in the biological activity, bioavailability, and solubility of nutrients (Ezhilarasi et al. 2013). Therefore, the T1:C1 sample could be more suitable for use in food. In this regard, there was a report about the effect of a further increase in the concentration of polymer and/or compounds on the increase in PS as well as the change in morphology. Thus, a smaller PS leads to better dissolution, probably due to the increased surface‐to‐volume ratio of nanoparticles (Pateiro et al. 2021). In one study, varying particle sizes were recorded for catechin encapsulated by lotus stem starch (615.6 nm), water chestnut (559.2 nm), and horse chestnut (322.7 nm), which is not consistent with our results. These differences in PS may be related to the type of stirring, emulsifier, and wall materials used (Ahmad et al. 2019).

### 3.3 Encapsulation Efficiency (EE) %

The encapsulation efficiency of the TP samples based on the amount of gallic acid obtained by HPLC is presented in Figure 1b. The results showed a significant increase in EE% in the T2:C1 sample (44.71 ± 0.15%). The EE percentage depends on the type of wall material and the interaction between the wall material and the core, the synthesis process, the PS, and the concentration of the core material (Chin, Mohd Yazid, and Pang 2014). Small particles exhibit enhanced EE% as a result of their capacity to create a suitable film around the surface of the core, thereby better preserving the encapsulated substance (Ahmad and Khan 2019; Zhu, 2017). This result is not consistent with our result. One of the reason may be related to the kind of wall material and its permeability. For the production of cocktail sausage, T2:C1 was chosen because it had the highest EE.

### 3.4 Bulk Density

For all the bead formulations, the BD did not have any significant difference. Powders with higher BD require less volume for packaging and vice versa.

The BD plays a crucial role in assessing its quality. Powders with higher density are typically selected for storage due to the reduced presence of air spaces between particles. This characteristic ultimately contributes to the oxidative reduction of core substances, thereby improving the stability of stored powdered encapsulates (Bashir et al. 2024).

### 3.5 Solubility Index

The solubility index in sample T1:C1 was greater (90.09 ± 0.23) than that in the other samples (*p* < 0.05) (Table 2). The solubility index decreased significantly with increasing CSM (*p* < 0.05). This feature indicates the capacity of the dried powder to form a solution or suspension in water. A higher solubility is preferred, mainly if the powder is used as an ingredient in the preparation of various products. Powder solubility is controlled by the quality of the raw materials, carrier material, moisture content, particle size, and physical state of the particles (Anisuzzaman et al. 2022; Tontul and Topuz 2017). Two samples, T2:C1 and T1:C1, showed higher solubility than T1:C2 (Table 2). In this regard, there is a report of high water solubility (above 87% w/w) in natural pigment samples of beet encapsulated with different wall materials (CSM, maltodextrin, and Arabic gum) (Antigo et al. 2020). Therefore, the wall material type and their combination may affect the SI. Moreover, a small PS creates a larger contact surface for interactions with water molecules, which leads to an increase in the SI (Medeiros et al. 2019).

### 3.6 Viscosity

**FIGURE 2:**
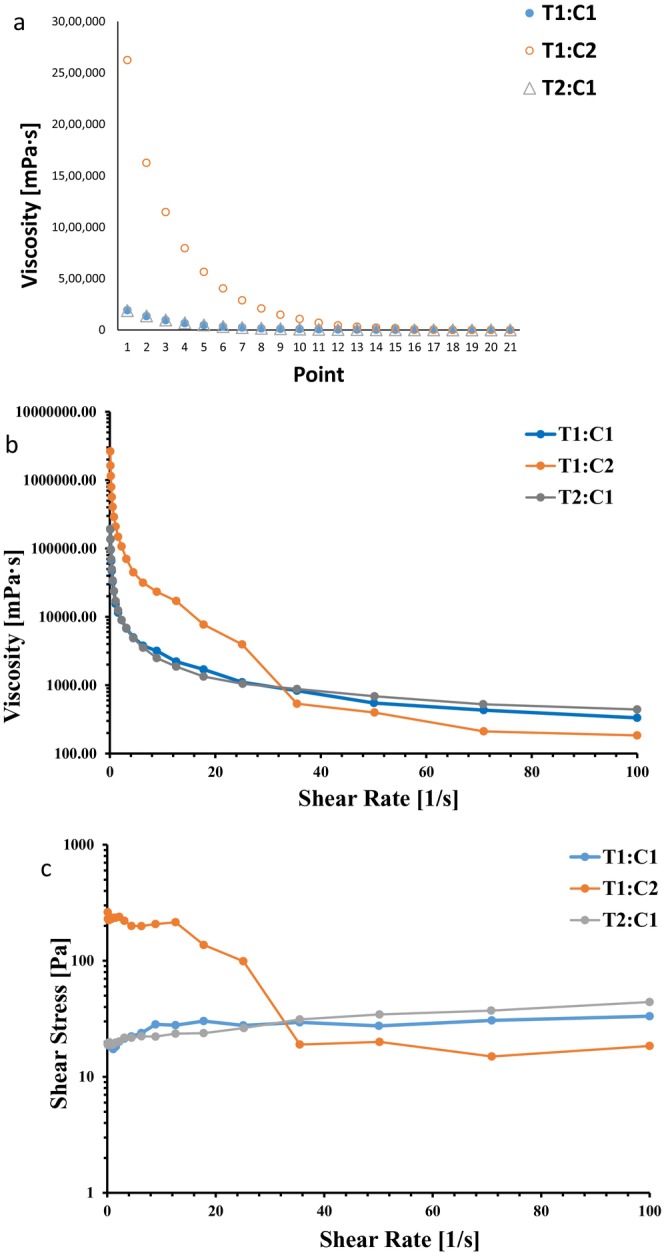
Relationship between viscosity (mPa/s) and shear rate (1/s) (a); point and viscosity (mPa/s) (b); shear rate (1/s) shear stress (Pa) (c). T1:C1, TP:CSM ratio in bead wall formulation were 1:1; T2:C1, TP:CSM ratio in bead wall formulation were 2:1; T1:C2, TP:CSM ratio in bead wall formulation were 1:2. CSM, chia seed mucilage; TP, tomato powder.

The viscosity of T1:C2 was the greatest among the samples (Figure 2a). In this study, the apparent viscosity increased proportionally to the concentration of CSM. This was attributed to the higher solid concentration, which led to the limitation of intermolecular movement based on hydrodynamic forces (Tamargo et al. 2018). The results showed that with increasing shear rate, the viscosity of the encapsulated samples decreased, which indicates that the samples exhibited relaxation behavior with time (pseudoplastic‐non‐Newtonian behavior) (Figure 2b).

As the shear rate continued to increase, the slope of the curve gradually decreased, indicating a decreasing trend in the viscosity of the liquid (Sekhavatizadeh et al. 2019). In the shear stress diagram based on the shear rate (Figure 2c), a distinct nonlinear relationship was observed across all the samples. Notably, T1:C2 exhibited a decreasing slope in the stress–shear diagram as the shear rate increased, indicating thixotropic behavior. Conversely, other samples displayed Bingham plasticity, suggesting a time‐dependent non‐Newtonian fluid where viscosity changes over time in response to applied force and shear stress.

### 3.7 X‐Ray Diffraction (XRD) Analysis

**FIGURE 3:**
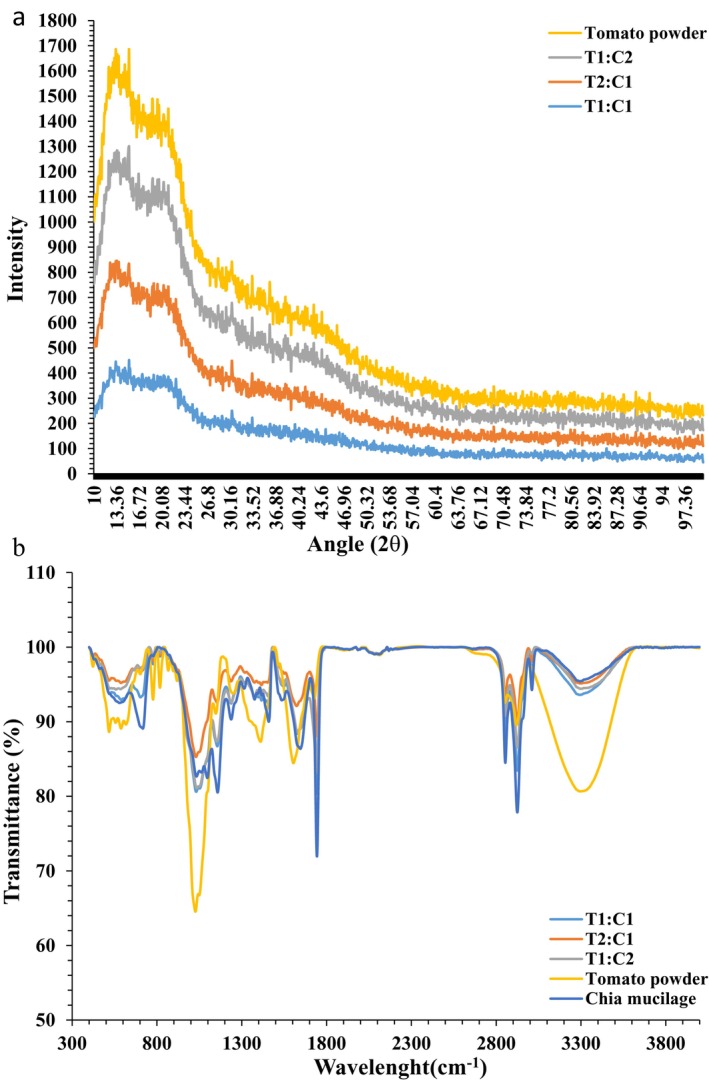
XRD (a) and FTIR (b) of microencapsulated TP with CSM. T1:C1, TP:CSM ratio in bead wall formulation were 1:1; T2:C1, TP:CSM ratio in bead wall formulation were 2:1; T1:C2, TP:CSM ratio in bead wall formulation were 1:2. CSM, chia seed mucilage; TP, tomato powder.

The X‐ray diffraction (XRD) patterns of the TP samples in free form and encapsulated with CSM are shown in Figure 3a. The XRD pattern shows a peak at approximately θ2 = 13.36 in all the samples, but a weak peak was observed only at approximately θ2 = 20 in the TP samples coated with CSM powder, which may be related to the amorphous structure (irregular structure) of the four samples.

The intensity of the peaks was somewhat greater for two samples, TP and T1:C2. In several studies, a crystalline peak has been identified in the same areas in CSM. The main crystalline peak was observed (θ2 = 14.77) in the pure CSM film. The peak was attributed to strong intermolecular and intramolecular hydrogen bonds. In addition, researchers reported a strong crystalline peak in the region of approximately θ3 = 15 for CSM (Fernandes et al. 2020). For TP, a peak (θ3 = 15) was observed without any diffraction. This could indicate a completely amorphous structure (irregular structure). Therefore, the two peaks observed for the encapsulated samples can be attributed to the presence of CSM with a semi‐crystalline structure. The semicrystalline structure of the beads increased with the microencapsulation process, which led to the removal of the amorphous structure of TP as a result of using CSM as a wall material in microencapsulation. In one study, it was observed that the nanoencapsulation of catechin samples by starch nanoparticles led to the production of nanoparticles with very amorphous characteristics (Ahmad et al. 2019), which is contrary to the results obtained in the present study. There may be several reasons for this difference. For example, the wall material type and microencapsulation method may affect the crystalline layer production. Raeisi et al. (2019) used different ratios of chitosan and Persian gum as wall materials for the microencapsulation of garlic essential oil. The results showed that the crystallinity index decreased with increasing Persian gum content in the wall materials. These results showed that the presence of Persian gum can increase the mobility of polymer chains and reduce crystalline structures by interfering with the molecular chain arrangement in chitosan (Raeisi et al. 2019).

### 3.8 Fourier Transform Infrared Spectroscopy

The functional groups in the TP, CSM powder, and TP coated with CSM powder were investigated by FTIR (Figure 3b). According to the chromatogram, four long peaks at wavenumbers 1027, 1744, 2853, 2925, and 3316 cm^−1^ were observed in the spectra of the TP and CSM powders, and the intensities of these peaks were reduced by microencapsulation. The broad bands observed at 3100–3600 cm^−1^ are related to O–H stretching with hydrogen bonding in the three encapsulated samples and TP. This band is specific to the functional groups of alcohols and phenols. All five investigated samples exhibited asymmetric stretching of H–C–H alkanes (2850–3000 cm^−1^). A long peak was observed for CSM (1744 cm^−1^), which decreased in intensity for all three encapsulated samples. Moreover, at 1648 cm^−1^, a peak was observed for CSM, and at 1629 cm^−1^, a peak attributed to the C=C stretching of the functional group (alkenyl) was observed for the three encapsulated samples. The intensity of the peaks decreased for the three encapsulated samples (Nandiyanto, Oktiani, and Ragadhita 2019). The observed peaks between 1470 and 11,350 cm indicate the H–C bending of alkanes (Ochida et al. 2019). At 1020–1250 cm^−1^, many peaks were observed in all five spectra, which are related to the C–N stretching state of aliphatic amines. In this range, a long peak related to tomato (1027 cm^−1^) was observed, and the intensity of this peak decreased in the three microencapsulation samples. Moreover, many weak peaks in the range of 675–900 cm^−1^ were observed in all five spectra related to the H–C aromatic ring (Nandiyanto, Oktiani, and Ragadhita 2019). Similar results were reported by Ochida et al. (2019) for fresh and dried tomato samples and by Goh et al. (2016) for CSM. Peaks at 1033 and 2910 cm^−1^ are usually found in polysaccharides, and the peaks at approximately 1420 cm^−1^ are usually found in gums; this characteristic gives anionic ions to the macromolecules (Goh et al. 2016; Ochida et al. 2019), which can confirm the results obtained from the spectroscopy of CSM powder in the present study.

### 3.9 Proximate, Cooking Losses, and Water Holding Capacity (WHC)

The physicochemical characteristics, including moisture, ash, fat, protein, WHC, pH, and cooking loss, are shown in Table 5. Compared with those in the Cont_sample_ treatment, the moisture content, fat, and cooking loss were significantly lower in the TP_sample_ and EnTP_sample_ (*p* < 0.05). The addition of the encapsulated form significantly decreased the fat content. The CSM can bind to water and decrease the moisture content. Moreover, the decrease in the moisture level of the products treated with TP and the encapsulated form can be attributed to the partial substitution of meat with low‐moisture compounds. The results of Surendar, Shere, and Shere (2018) were in line with our research. In the present study, the increase in ash content in fortified sausage samples can be attributed to the high ash content of TP (10.78% w/w) and CSM powder (8.71% w/w) (Surendar, Shere, and Shere 2018; Tavares et al. 2018). The amount of protein was equal among the samples. In this study, TP was added to the sausage formulation at 3% w/w and with flaxseed powder (6% w/w), which caused a significant increase in the amount of protein. The findings of this research are not consistent with our study. One of the reasons could be the amount and type of additives used in the formulation (Ghafouri‐Oskuei et al. 2020).

**TABLE 5:** Proximate analyses of cocktail sausage.

Proximate value (%w/w)	EnTP_sample_	TP_sample_	Cont_sample_
Moisture	31.65 ± 0.22 c	34.74 ± 0.19 b	41.85 ± 0.22 a
Ash	5.15 ± 0.26 a	4.87 ± 0.15 a	4.42 ± 0.13 b
Fat	17.20 ± 0.51 c	25.88 ± 0.33 b	27.33 ± 0.44 a
Protein	32.65 ± 0.31 a	31.52 ± 0.28 b	31.24 ± 0.3 b
WHC	6.92 ± 0.15 a	5.66 ± 0.19 b	4.79 ± 0.16 c
Cooking losses	10.98 ± 0.57 b	11.12 ± 0.92 b	15.43 ± 0.96 a

*Note:* Values are expressed as mean ± SD. Data (mean ± standard deviation) are from three replications. Means followed by different lowercase letters in row differ (*p* < 0.05) by Duncan test.

Abbreviations: Cont_sample_, control sample; dw, Dry weight; EnTP_sample_, encapsulated tomato powder sample were used in cocktail sausages; TP_sample_, tomato powder sample.

Adding TP as free and encapsulated forms to sausage led to an increase in WHC and a decrease in cooking loss. The results were obtained from the findings of Saengphol and Pirak (2019), who reported that a frankfurter sausage sample containing spicy basil seed mucilage (*Ocimumcanum Sims*.) had a lower water holding capacity and cooking loss than the control sample (Saengphol and Pirak 2019). These results are consistent with our results. One of the reasons for the increase in WHC in the enriched sausage samples was the type of ingredient. CSM is a polysaccharide with a fiber‐rich component that consists of several free hydroxyl groups that can bond with water molecules, increasing the WHC (Punia and Dhull 2019).

### 3.10 Texture and Color

The TPA results, including the hardness, adhesiveness, cohesiveness, springiness, gumminess, and chewiness of the sausage samples on the first and 40th days of storage, are shown in Table 6. The TPA results showed that increasing the storage time caused an increase in all the texture parameters in the sausage samples. A similar result was reported by Fu et al. (2022). The hardness of the Cont_sample_ exhibited a notable increase (*p* ≤ 0.05) over the duration of storage (Table 6). It is believed that the rise in hardness observed in meat products during refrigeration is attributed to the destabilization of the emulsion, which occurs due to the separation of water and fat from the protein matrix (Estévez, Ventanas, and Cava 2005). Protein oxidation can contribute to an increase in hardness by facilitating the formation of carbonyl compounds, resulting in a decline in protein functionality and promoting the establishment of protein cross‐linking through disulfide bonds (Ganhão, Morcuende, and Estévez 2010). The Cont_sample_ in this study also exhibited a greater increase in lipid oxidation, which may be one of the reasons for the increased firmness of the texture during the storage period (Table 8). The results of this study indicated that the addition of TP inhibits the increase of hardness of sausages during refrigerated storage. In a previous study, Estévez, Ventanas, and Cava (2006) demonstrated that incorporating 0.1% sage extract led to a significant decrease in the hardness of porcine liver pâté after 30 days of refrigeration. This finding suggests that natural antioxidants can play a crucial role in reducing the hardness of meat products by enhancing emulsion stability and minimizing cross‐linking, primarily through their protective effects on proteins against oxidative damage. Furthermore, these natural antioxidants may safeguard muscle membranes from lipid oxidation, thereby preserving the integrity of muscle fiber membranes and reducing moisture loss, which ultimately contributes positively to the textural characteristics of sausages (Maqsood, Benjakul, and Balange 2012). The changes of gumminess and chewiness of sausages during refrigerated storage were similar to the hardness. None of the treatments had any effect on the springiness and cohesiveness values of the sausages during storage compared to the control. These results suggest that sage can minimize texture deterioration of sausage caused by oxidation (Zhang et al. 2013).

**TABLE 6:** Texture parameters of cocktail sausages during storage time.

Texture parameters	Day	EnTP_sample_	TP_sample_	Cont_sample_
Hardness (g)	1	2022.7 ± 107.10 abA	2561 ± 409.98 aA	1512.7 ± 209.37 bB
	40	2992.5 ± 153.85 aA	2233.2 ± 121.16 bA	2225.5 ± 162.27 bA
Adhesiveness (mJ)	1	0.10 ± 0.05 bB	0.10 ± 0.04 bB	0.36 ± 0.11 aB
	40	0.42 ± 0.21 bA	0.44 ± 0.09 bA	0.80 ± 0.12 aA
Cohesiveness	1	0.80 ± 0.04 aB	0.81 ± 0.01 aB	0.85 ± 0.05 aB
	40	1.06 ± 0.06 aA	1.04 ± 0.13 aA	1.20 ± 0.15 aA
Springiness (mm)	1	5.73 ± 0.33 aB	5.64 ± 0.39 aB	5.85 ± 0.08 aB
	40	7.29 ± 0.37 aA	7.80 ± 0.96 aA	8.10 ± 1.49 aA
Gumminess (g)	1	1659.2 ± 74.55 bA	2108.5 ± 343.62 aB	1351.1 ± 84.24 bB
	40	2772.3 ± 774.72aA	1882.1 ± 142.99 bA	1665.6 ± 34.76 cA
Chewiness (mJ)	1	94.09 ± 90.56 abB	114.39 ± 26.94 aA	77.95 ± 5.84 bA
	40	125.37 ± 5.32 aA	137.21 ± 17.53 aA	95.24 ± 13.88 bA

*Note:* Data (mean ± standard deviation) are from three replications. Means followed by different lowercase letters in rows and uppercase letters in columns differ (*p* < 0.05) by Duncan test.

Abbreviations: Cont_sample_, control sample; EnTP_sample_, encapsulated tomato powder sample were used in cocktail sausages; TP_sample_, tomato powder sample.

The CSM may interfere in some way with the molecular linkage of the emulsified meat matrix. Additionally, in research, the hardness and chewiness of all the meat samples containing CSM gel increased, which may be due to the high water holding capacity, high fiber content, and high thickening properties of CSM (Câmara, Okuro et al. 2020; Gao et al. 2024).

The EnTP_sample_ had the most significant difference in all parameters except adhesiveness, cohesiveness, and springiness at the same time (*p* ≥ 0.05). Lee and Chin, Mohd Yazid, and Pang (2014) reported that the addition of basil seed gum as a fat replacer to low‐fat sausage led to an increase in gumminess but did not increase cohesiveness or springiness compared to the control sample (Lee and Chin 2017). These results are consistent with the results obtained in this study.

The results of the texture analysis may be attributed to the fact that the addition of CSM and TP could increase the viscosity of the meat batter and promote hydrogen bonding between CSM and meat proteins, which promoted the formation of a compact network structure with the meat proteins and improved the textural properties of the sausage (Cao et al. 2022).

The EnTP _sample_ had the lowest L* value among the samples (Table 7). The primary cause for the change in the color of sausages may be related to the brownish color of CSM (a natural pigment in chia seeds). Similarly, a CSM brownish color was also recorded by Câmara, Geraldi et al. (2020) (Câmara, Okuro et al. 2020; Gao et al. 2024).

**TABLE 7:** Color parameters of cocktail sausages during storage time.

	Storage time	EnTP_sample_	TP_sample_	Cont_sample_
L*	1	38.89 ± 12.05^aA^	48.78 ± 6.45^aB^	43.67 ± 5.14^aB^
	10	44.78 ± 8.31^bA^	47.67 ± 12.98^bB^	58.67 ± 10.18^aA^
	20	43.78 ± 7.2^aA^	44.56 ± 6.30^aB^	52.11 ± 14.69^aAB^
	30	45.00 ± 1.93^cA^	56.89 ± 2.62^aA^	50.78 ± 2.77^bAB^
	40	44.00 ± 2.23^cA^	55.11 ± 2.66^aA^	48.89 ± 1.16^bB^
a*	1	11.89 ± 1.83^bB^	19.22 ± 7.17^aA^	8.87 ± 0.86^bB^
	10	13.78 ± 2.10^bA^	19.56 ± 5.24^aA^	10.89 ± 3.75^bA^
	20	14.33 ± 2.39^bA^	21.22 ± 6.2^aA^	11.44 ± 2.4^bA^
	30	4.56 ± 0.52^bC^	8.78 ± 2.72^aB^	4.44 ± 0.72^bC^
	40	4.00 ± 0.70^bC^	7.00 ± 1.22^aB^	4.22 ± 0.97^bC^
b*	1	24.89 ± 2.35^bB^	29.89 ± 4.42^aB^	15.22 ± 2.16^cD^
	10	29.22 ± 2.90^aA^	29.67 ± 3.80^aB^	24.56 ± 2.35^bB^
	20	27.78 ± 2.77^bAB^	33.67 ± 5.91^aA^	21.44 ± 3.77^cC^
	30	15.22 ± 1.20^cC^	40.56 ± 0.52^bC^	45.00 ± 1.93^aA^
	40	14.78 ± 1.78^cC^	40.56 ± 1.13^bC^	45.89 ± 2.97^aA^

*Note:* Data (mean ± standard deviation) are from three replications. Means followed by different lowercase letters in rows and uppercase letter in columns differ (*p* < 0.05) by Duncan test.

Abbreviations: Cont_sample_, control sample; EnTP_sample_, encapsulated tomato powder sample were used in cocktail sausages; TP_sample_, tomato powder sample.

The enhancement of L* in EnTP_sample_ and Cont_sample_ were not significant but it was increased in TP_sample_. The type of packaging, particularly in relation to oxygen availability, could play a significant role in influencing changes that occur during storage. The results obtained from this research are consistent with the findings of Jo, Lee, and Ahn (1999). The a* value of all samples gradually decreased as storage time increased. This means loss of redness in the color of the meat and the transition of its color to brownish red by the formation of metmyoglobin (Tangkham and LeMieux 2017). In this research, the decrease in a* aligns with the findings of Kulkarni et al. (2011). The b* increased in TP_sample_ and Cont_sample_ but decreased in EnTP_sample_ during storage. In agreement with my findings, b* decreased (*p* < 0.05) during storage time in fresh pork sausage containing chia seed (*S. hispanica*) as an antioxidant. The b* parameter decreased over storage time in the EnTP_sample_ because this treatment did not undergo oxidation during storage (Scapin et al. 2015).

During the storage period, TP_sample_ had the highest a* value (*p* < 0.05). A similar study was conducted by Ghafouri‐Oskuei et al. (2020) on the addition of flax seed powder and TP to cooked sausages, which caused an increase in the a* and b* values. This can be attributed to the presence of lycopene in the TP (Ghafouri‐Oskuei et al. 2020), which is consistent with the results of the present study.

The a* was the lowest in the EnTP _sample_ on the 40th day of storage. In similar research, adding CSM to Bologna sausages decreased a* values (Câmara, Geraldi et al. 2020).

### 3.11 Peroxide Value and Thiobarbituric Acid‐Reactive Substances

The PV and TBARS of the sausage samples are presented in Table 8. The assessment of lipid oxidation is predominantly conducted through the detection of oxidation products at both the primary and secondary stages. Primary lipid oxidation products are typically measured using PV, whereas the evaluation of secondary lipid oxidation is primarily carried out through the TBARS assay. This method is particularly effective in quantifying malondialdehyde, a key compound generated during lipid oxidation, thereby serving as an important indicator of food quality and the extent of oxidative deterioration (Zhao et al. 2020). In this research, these parameters increased significantly during the storage period (*p* < 0.05). The amount of PV in the samples increased from 1.34–1.36 mEq O_2_/kg to 7.34–16.79 mEq O_2_/kg. The same changes were observed in the TBARS parameters (0.97–0.99 mg malondialdehyde/kg to 2.09–3.46). In storage time, temperature, presence of light, and oxygen increase oxidative processes. Additionally, the use of other ingredients (antioxidants) in meat product formulations also influences the oxidation process. Besides heat, pH, light, oxygen, oxidation time, packaging, type of buffer, water activity, the form of substrate, and the presence of unsaturated fatty acids are the major factors affecting lipid oxidation (Domínguez et al. 2019).

**TABLE 8:** Lipid oxidation parameters of cocktail sausages during storage time.

Teatments	TBARS (mq MDA/kg sample)		PV (mEq/kg)	
	Day 1	Day 40	Day 1	Day 40
Cont_sample_	0.97 ± 0.09 bA	3.46 ± 0.22 aA	1.36 ± 0.06 bA	16.79 ± 0.59 aA
TP_sample_	0.98 ± 0.05 bA	2.47 ± 0.12 aB	1.34 ± 0.05 bA	9.47 ± 0.99 aB
EnTP_sample_	0.99 ± 0.11 bA	2.09 ± 0.14 aC	1.35 ± 0.09 bA	7.34 ± 0.43 aC

*Note:* Data (mean ± standard deviation) are from three replications. Means followed by different lowercase letters in row and uppercase letter in column differ (*p* < 0.05) by Duncan test.

Abbreviations: Cont_sample_, control sample; EnTP_sample_, encapsulated tomato powder sample were used in cocktail sausages; MDA, malondialdehyde; TBARS, thiobarbituric acid reactive subestance; TP_sample_, tomato powder sample.

The findings of the present study showed that the Cont_sample_ exhibited the highest level of PV and TBARS, whereas EnTP_sample_ displayed the lowest index of lipid oxidation on the 40th day of storage and the TP_sample_ had lower lipid oxidation index than the Cont_sample_. Natural antioxidants from plant by‐products are increasingly used in meat products to reduce oxidative changes during storage by donating hydrogen atoms to free radicals and either reacting with oxygen or scavenging metal ions that catalyze oxidation (Hadidi et al. 2022). In this study, the presence of phenolic compounds in CSM and TP is responsible for their antioxidant activity. In this regard, a study conducted by Teets and Were (2008) reported that the addition of almond skin powder (10% w/w) to chicken sausage samples delayed the production of primary lipid oxidation products. Our research is consistent with these findings (Teets and Were 2008). Jouki, Rabbani, and Shakouri (2020) reported that flavonoids and phenolic acids are responsible for the high antioxidant activity of tomatoes. These materials are sensitive to heat, so they decrease in quantity in meat products during pasteurization and lead to decreased antioxidant properties (Jouki, Rabbani, and Shakouri 2020). An alternative solution to this problem is the implementation of microencapsulation. Encapsulation protects natural antioxidants by enclosing them in wall materials, preventing direct contact with food components. Therefore, the antioxidant content of encapsulated bioactive material (TP) was more stable than TP alone. Moreover, EnTP_sample_ consists of two phenolic compounds (CSM and TP) in comparison to TP_sample_. Numerous research efforts have focused on the encapsulation of components derived from plant extracts, aiming to incorporate these substances as functional ingredients within food matrices and in the development of food products (Hadidi et al. 2022). For example, the application of *Zanthoxylum bungeanum* essential oil microcapsules in Chinese‐style sausage effectively inhibited lipid oxidation in sausages and protected typical volatiles from volatilization and degradation during sausage storage (Meng et al. 2022).

### 3.12 Sensory Evaluation of Sausage

**FIGURE 4:**
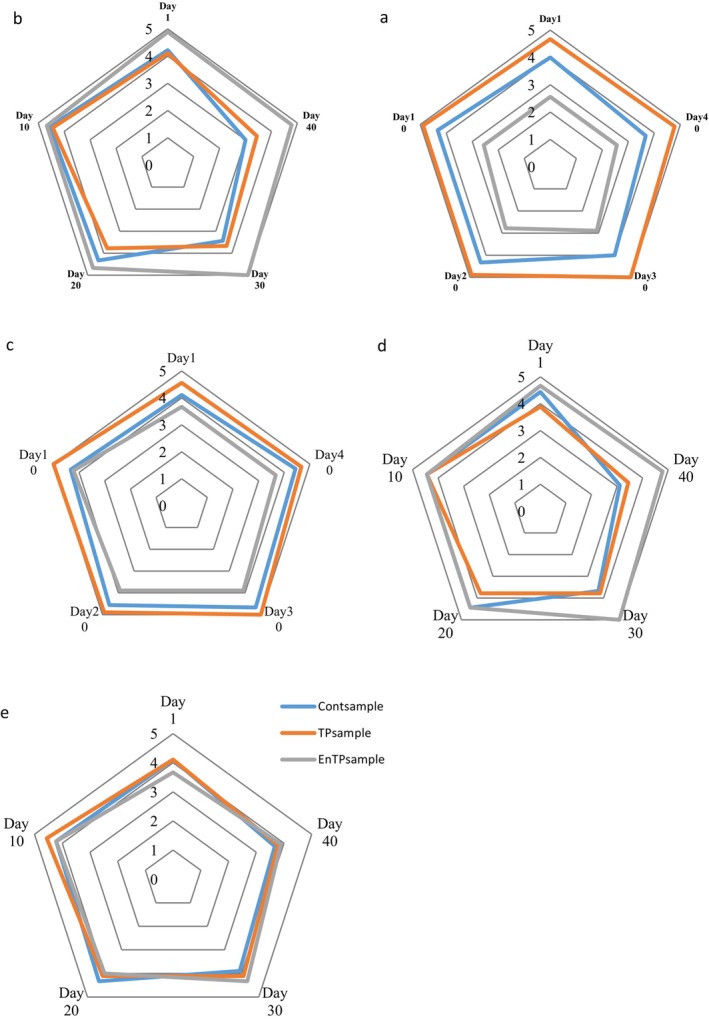
Sensory properties of cocktail sausages during storage time. Sensory parameters consist of texture (a), color (b), flavor (c), odor (d), and acceptability (e). Cont_sample_, control sample; EnTP_sample_, encapsulated tomato powder sample; TP_sample_, tomato powder sample.

The results of sensory characteristics, including texture, odor, color, taste, and overall acceptance, are shown in Figure 4. The odor was reduced in all samples during storage. Oxidation results in the generation of secondary oxidation products, which play a significant role in the development of unpleasant odors. These products include various aldehydes, such as n‐alkanals, trans‐2‐alkenals, 4‐hydroxy‐trans‐2‐alkenals, and malondialdehyde (Schilling et al. 2019). The rate of odor score reduction (1.5 scores) in Cont_sample_ was greater than the others. One of the reasons may be related to the antioxidant properties of TP and CSM. They reduced the rate of oxidation so off‐odor compounds were produced at a low rate in comparison to Cont_sample_. The flavor was constant among the samples except Cont_sample_ that was increased. One of the reasons may be related to TP and CSM antioxidant properties because they reduced volatile compound production. The EnTP_sample_ was constant in color but TP_sample_ and Cont_sample_ were reduced during the storage time. These results can be due to the antioxidant effect of TP and CSM in color stability of sausage, since in Cont_sample_, aldehyde products formed during lipid oxidation covalently bind to myoglobin and accelerate heme oxidation and metmyoglobin formation, leading to meat discoloration (de Almeida et al. 2015). Texture and total acceptability scores of Cont_sample_, TP_sample_, and EnTP_sample_ were constant during the storage time.

The results demonstrate that the TP_sample_ had a valuable taste, color, and overall acceptability compared to the other samples. However, the EnTP_sample_ had the highest score for texture and smell parameters. Consistent with the current research, So, Uriyapongson, and Uriyapongson (2020) used tomato waste (1%–5% w/w) in frankfurter sausage. The results showed that sausages containing 2% w/w powder had a higher score in terms of taste, texture, appearance, and overall acceptability than the control sample (So, Uriyapongson, and Uriyapongson 2020). One of the reasons for the difference in the results obtained from the current study is the variance in the percentage of TP used compared to the current research. The addition of pure TP caused a favorable and pinkish color in the sausage samples, while the fine‐coated TP led to the creation of a dark gray color in the product, which was probably due to the presence of CSM powder in the microencapsulation. The presence of CSM powder in the microcapsules created a favorable gel texture in the product, which led to a high score for this parameter.

## 4 Conclusion

In this study, free and encapsulated TP were used in sausage formulations. It seemed that greenness and blueness were the most influential factors. In this study, the PV assay showed that the encapsulated form had extensive antioxidant activity. In addition, the TBARS results indicated the strong antioxidant activities of the encapsulated TP. The sensory results demonstrated that the encapsulated TP samples had the greatest color and odor value during storage. It can be concluded that the encapsulation of TP increased the hardness and gumminess. Furthermore, CSM can be recommended as a natural wall material due to its good coating characteristics, which are useful for encapsulating TP and supplementing cocktail sausage.

## Author Contributions

**Seyed Saeed Sekhavatizadeh:** writing – original draft (equal), writing – review and editing (equal). **Fatemeh Faryabi:** investigation (lead), methodology (lead). **Mohammad Ganje:** investigation (equal).

## Ethics Statement

The authors have nothing to report.

## Consent

The authors have nothing to report.

## Conflicts of Interest

The authors declare no conflicts of interest.

## Data Availability

Data that support the findings of this study are available upon reasonable request.
